# Combined transcriptomics and metabolomics analysis reveals the molecular mechanism of heat tolerance of Le023M, a mutant in *Lentinulaedodes*

**DOI:** 10.1016/j.heliyon.2023.e18360

**Published:** 2023-07-17

**Authors:** Qin Zhang, Rencai Feng, Renyun Miao, Junbin Lin, Luping Cao, Yanqing Ni, Wensheng Li, Xu Zhao

**Affiliations:** a Institute of Urban Agriculture, Chinese Academy of Agricultural Sciences, Chengdu, 610299, Sichuan, China; b Chengdu National Agricultural Science and Technology Center, Chengdu, 610299, Sichuan, China; c College of Life Science and Technology, Gansu Agricultural University, Lanzhou, 730070, Gansu, China; d College of Food and Biological Engineering, Chengdu University, Chengdu, 610106, Sichuan, China; e Facility Agriculture and Equipment Research Institute, Gansu Academy of Agri-engineering Technology, Wuwei, 733006, Gansu, China

**Keywords:** *Lentinula edodes*, Heat stress, Protein folding, Metabolite, Trehalose

## Abstract

*Lentinula edodes*, one of the most highly regarded edible mushrooms in China, is susceptible to damage from high temperatures. However, a mutant strain derived from *L. edodes*, known as Le023M, has shown exceptional thermotolerance. Compared to the original strain Le023, Le023M exhibited accelerated mycelial recovery following heat stress. Through RNA-seq analysis, the majority of differentially expressed genes (DEGs) were found to be associated with functions such as “protein refolding”, “protein unfolding”, “protein folding”, and “response to heat”, all of which are closely linked to heat shock proteins. Furthermore, qRT-PCR results revealed significant accumulation of heat shock-related genes in Le023M under heat stress. GC-MS analysis indicated elevated levels of trehalose, aspartate, and glutamate in Le023M when subjected to heat stress. The highly expressed genes involved in these metabolic pathways were predominantly found in Le023M. Collectively, these findings highlight the following: (i) the crucial role of heat shock proteins (HSPs) in the thermo-resistant mechanisms of Le023M; (ii) the potential of trehalose accumulation in Le023M to enhance mycelium resistance to heat stress; and (iii) the induction of aspartate and glutamate accumulation in response to heat stress. These results shed light on the molecular mechanisms underlying the thermotolerance of Le023M, providing valuable insights for further understanding and improving heat stress response in *L. edodes*. The findings also highlight the potential applications of Le023M in the cultivation and production of *L. edodes* under high-temperature conditions.

## 1 Introduction

*Lentinula edodes*, which is known as the shiitake mushroom and is an edible mushroom in East Asia. It holds the distinction of being the major edible fungus, accounting for 22% of the world’s cultivated mushrooms [1]. With its low fat content, *L. edodes* is rich in polyunsaturated fatty acids, constituting over 90% of its composition [2]. Beyond its culinary appeal, *L. edodes* is highly regarded for its health benefits, thanks to active compounds like lentinan. Lentinan exhibits antimicrobial, anti-cancer, liver-protective, anti-diabetic, anti-flu, and anti-leukemia properties [[3], [4], [5], [6], [7]]. The agricultural cultivation and biotechnological applications of *L. edodes* have had a profound positive impact on people’s lives, owing to its valuable properties. In fact, China alone accounted for 90% of global production, yielding an estimated 11.64 million tons of *L. edodes* in 2020 (https://www.chyxx.com/industry/202201/992805.html). The cultivation of *L. edodes* traces its roots back to China 800 years ago.

Growth and development of edible fungi, including *L. edodes*, are significantly influenced by environmental factors, such as high temperatures. When L. *edodes* is cultivated at high temperatures, it experiences an increase in cell membrane fluidity, leading to structural changes in organelles and a decrease in enzyme activity [8]. Mycelium development is negatively affected when temperatures exceed 32 °C, resulting in poor mycelia. Temperatures above 35 °C can even cause severe growth retardation or death. During the warmer months, the viability of spring-inoculated *L. edodes* mycelia deteriorates, rendering them vulnerable. Moreover, at high temperatures, the culture substrates or bags are more susceptible to pathogen infections, leading to reduced yields and compromised quality [9,10].

Based on transcriptomic, proteomic, and metabolomic analysis, the pattern of molecular expression of *L. edodes* in response to heat stress from mRNA, protein, and metabolites can be analyzed. This allows for a more comprehensive understanding of *L. edodes* molecular mechanisms in response to heat stress. It also provides insight into the development of strategies to improve its resistance to heat stress. The ion channel protein, G protein-coupled receptor, and histidine kinase are the first signal receptors to detect heat stress signals. Second messengers such as Ca^2+^, ABA, and phosphoglycerol can then activate transcription factors. Then, these TFs activate the target genes involved in heat stress to produce a variety of protective proteins for cells, including heat stock proteins (HSPs) and late embryogenesis abundant (LEA) proteins [11,12]. HSPs serve as molecular chaperones and are synthesized in response to high temperatures, salinity, drought, starvation, and heavy metal ions [13]. In abiotic stress, HSPs work as molecular chaperones, facilitating the refolding, stabilization, and assembly of other proteins without being part of a final structure [14]. Trehalose is produced as a metabolite in response to heat stress. Heat stress induces endogenous trehalose accumulation in *Pleurotus eryngii* [15]. Additionally, the supplementation of 100 or 200 g/L trehalose significantly inhibits the production of thiobarbituric acid-reactive substance (TBARS) in mycelial cells, providing cellular protection against superoxide toxicity during heat stress.

One specific *L. edodes* strain, Le023M, was selected from the protoplasts of the original strain Le023, which had undergone UV treatment [16]. Le023M was subjected to a heat shock at 37 °C for 24 h in potato dextrose (PDA) medium to evaluate its thermotolerance capacity and recovery rate. By conducting a comprehensive analysis of transcription and metabolism levels using integrated transcriptomic and metabolomic approaches, the mechanisms underlying heat stress were elucidated. This analysis provided valuable insights into the molecular expression patterns associated with heat stress responses. In response to heat stress, differentially expressed genes (DEGs) and differentially metabolites were identified and mapped into potential regulatory biological pathways. These pathways were crucial for the adaptation of the organism to its thermal environment. The findings from this study not only enhanced our understanding of the molecular foundations of heat stress but also paved the way for the discovery of previously unknown genes and pathways associated with heat response. This knowledge can be leveraged to develop strategies aimed at enhancing heat tolerance in crops and other organisms.

## 2 Materials and methods

### 2.1 Sample information

Le023M, derived from the main cultivar of *L. edodes* in Hebei, Le023, is a mutant strain. Its authenticity was verified using an inter-simple sequence repeat (ISSR) marker, and it exhibited enhanced characteristics such as rapid growth and a pleasant taste [17]. The culture technique findings demonstrated that Le023M exhibited a swift recovery from heat stress and promptly initiated the fruiting process.

### 2.2 Cultured and heat stress treatment

**Fig. 1:**
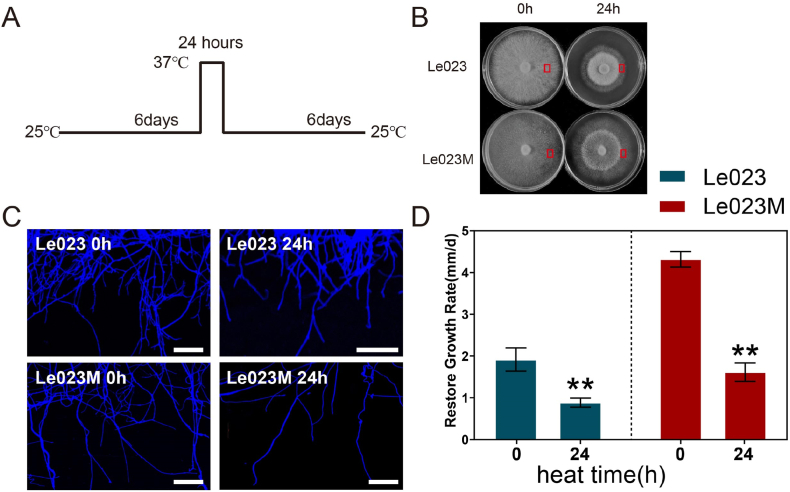
Response to Heat Stress in Two Tested *L. edodes* Strains. (A) Culturing conditions for two strains. The mycelia were cultured on PDA medium at 25 °C for 6 days, followed by exposure to 37 °C for 0 or 24 h, and then cultured at 25 °C for an additional 6 days. (B) Phenotype of mycelia exposed to 37 °C for 0 and 24 h. (C) Observation of mycelia undergoing heat stress for 0 or 24 h. (D) Recovery of mycelial growth after heat stress in *L. edodes*. The values presented are the means ± standard deviation (SD) of three independent experiments. Statistical significance is denoted as **p < 0.01.

Le023 and Le023M mycelia were taken out from 4 °C and activated three times on PDA medium. A 1 cm^2^ section of mycelia-medium mixture was obtained by punching and transferred to a new PDA plate. The plate was then incubated for six days at 25 °C. Afterwards, the mycelia were cultured at 37 °C for 0 and 24 h (Fig. 1A). In order to obtain optimal results, we conducted temperature screening and found that Le023 and Le023M exhibited the largest phenotypic difference at 37 °C for 24 h. Therefore, we selected 37 °C as the treatment temperature (Fig. S1). Following that, all samples were transferred back to 25 °C and cultured for an additional 6 days. After the cultivation period, photos were taken of the samples.

Trehalose was added to the PDA medium until the appropriate final concentrations of 0, 5, 10, and 20 g/L were achieved. The medium was then sterilized at 121 °C for 20 min and cooled back down to 60 °C. Subsequently, the medium was separated into sterilized petri dishes. The heat treatment of mycelia was performed in the same manner.

For microcopy analysis, a coverslip was inserted diagonally into a fresh PDA medium, positioned 3 cm away from the center of the Petri dish. Following incubation at 25 °C, allowing the mycelia to grow and spread onto the coverslip, the samples were subjected to a heat shock by transferring them to 37 °C for either 0 h (control) or 24 h. After the heat treatment, both Le023 and Le023M mycelia were incubated at 25 °C for additional six days, until the coverslip was fully colonized. Subsequently, a glass slide was placed on one side of the mycelia, which were then observed using a Leica STELLARIS 5 Confocal Microscopy (Leica Microsystems, Wetzlar, Germany). The resulting images were processed using Photoshop CS6 software. This approach allowed for detailed observation of the mycelial structures and collection of the required data.

The activated mycelia were homogenized with medium using a homogenizer and then transferred into a triangular flask containing 100 mL of potato dextrose broth (PDB) medium. The cultivation process took place at 25 °C with agitation at a speed of 150 rpm for 7 days. Subsequently, 10 mL of the fungal endophyte culture supernatant was transferred to a new flask containing 100 mL of fresh PDB medium. This new mixture was further incubated under the same conditions, 25 °C and 150 rpm, for an additional 14 days. At the end of the 14-day period, the endophyte culture had reached a sufficient quantity for harvesting. The biomass was then separated from the broth and stored at −80 °C for further analysis.

### 2.3 Relative electric conductivity (REC) test

Relative electric conductivity of the samples, abbreviated as REC, was measured as described in rice [18]. Mycelia samples with different times of heat stress were placed into 50-mL tubes containing 30 mL of deionized water. The tubes were vibrated occasionally at 25 °C for 30 min, and then the electrical conductivity of the mixture was measured as **E1**. As a control, a blank consisting of only deionized water without sample (**E0**) was measured. After boiling the samples for 30 min, the electrical conductivity was measured as **E2**. The difference between E1 and E2 was used to determine the REC value.

### 2.4 Malondialdehyde (MDA) content

The content of MDA was detected following the method of Heath and Packer [19] with some modifications. Mycelia taken out from −80 °C were freeze-dried and ground into a fine powder. Triplicate 20 mg aliquots were extracted in 1 mL 10% of trichloroacetic acid (TCA) for 30 min at 4 °C. After centrifugation at 12,000*g* for 10 min, 0.2 mL of supernatant was transferred to a new 2 mL screwed centrifuge tube containing 0.8 mL of 0.67% of thiobarbituric acid (TBA). The mixture was boiled for 20 min. After centrifugation at 12,000*g* for 10 min, 300 μl aliquots in triplicates were transferred to 96-well flatted bottom plate. Absorbance was read at 532 nm and 600 nm, respectively. The content of MDA was calculated as follows: MDA content (nmol/g) = 53.763 × ***ΔA*** ÷ **W** (***ΔA*** was difference of *ΔA*_532_ and ΔA_600_. **W** was weight of mycelia).

### 2.5 H_2_O_2_ content

The hydrogen peroxide (H_2_O_2_) content was determined using the starch hydrogen peroxide assay kit (Megazyme) in a 96-well plate format. The assay was adapted from the manufacturer’s protocol, and appropriate dilutions were made. The results were expressed as the mean of three technical replicates, which were then displayed.

### 2.6 Measurement of ascorbate (AsA) and glutathione (GSH and GSSG)

The AsA kit (Nanjing Jiancheng Bioengineering Institute, China) was used to determine the AsA content in accordance with the manufacturer’s instructions. The kit contains all the necessary reagents, including the AsA standard, for the AsA assay.

GSH and GSSG were extracted from samples and the contents were measured using GSH kit and GSSG kit (Beyotime, China) according to the manufacturer’s instructions. The extraction process was carried out strictly following the guidelines provided by the manufacturer.

### 2.7 RNA extraction and library preparation

Total RNA was extracted using the MagMAX™ mirVana™ isolation kit (Thermo Fisher), following the protocol of the manufacturer. RNA integrity was evaluated using the Agilent 2100 Bioanalyzer (Agilent Technologies, Santa Clara, CA, USA). Samples with RNA Integrity Number (RIN) ≥ 7 were subjected to subsequent analysis. The libraries were constructed using TruSeq Stranded mRNA LTSample Prep Kit (Illumina, San Diego, CA, USA) according to the manufacturer’s instructions. Then, these libraries were sequenced on the Illumina sequencing platform (HiSeqTM 2500), and 125 bp/150 bp paired-end reads were generated.

### 2.8 RNA-seq data analysis

Raw data (raw reads) were processed using Trimmomatic [20]. The reads containing ploy-N and low-quality reads were removed to obtain the clean reads. After removing the adaptor and low-quality sequences, the clean reads were assembled into expressed sequence tag clusters (contigs) and de novo assembled into transcript by using Trinity [21] (version: trinityrnaseq_r20131110) in paired-end method. The longest transcript was chosen as an unigene based on the similarity and length of a sequence for subsequent analysis.

The function of the unigenes was annotated by alignment with the NCBI non-redundant (NR), SwissProt, and clusters of orthologous groups for eukaryotic complete genomes (KOG) databases in Blastx [22] with a threshold E-value of 10^−5^. The proteins with highest hits to the unigenes were used to assign functional annotations. Based on the SwissProt annotation, GO classification was performed based on the mapping relationship between SwissProt and GO term. The unigenes were mapped to KEGG [23] database to annotate their potential metabolic pathways. FPKM [24] and read counts value of each unigene was calculated using bowtie2 [25] and express [26]. DEGs were identified using the DESeq package in Rstudio (version 4.2.1). Data were normalized using the function of estimate size Factors. P value and foldchange were determined using the function of nbinom Test. P value < 0.05 and foldChange > 2 or foldChange < 0.5 were set as the threshold for significant differential expression. Hierarchical cluster analysis of DEGs was performed to explore the transcript expression pattern. GO enrichment and KEGG pathway enrichment analysis of DEGs were performed using Rstudio (version 4.2.1) based on the hypergeometric distribution. Heatmap was generated using the pheatmap package in Rstudio (version 4.2.1).

### 2.9 Validation of RNA-seq data by qRT-PCR

Quantitative reverse transcription-PCR (qRT-PCR) was performed to measure gene expression, following the procedure described by Mieog, Janeček [27]. The *TUB* [28] gene was selected as the reference gene for normalization. The target gene expression was determined by the *E*^−▵▵Ct^ method against the Ct value of TUB. The qRT-PCR primers used are listed in Table S1.

### 2.10 Metabolome analysis

Sample preparation, measurement, and analysis were performed as described by Zhao, Chen [29]. The Agilent 7890B gas chromatography system, in conjunction with the Agilent 5977A MSD system (Agilent Technologies Inc., CA, USA), was used to analyze the derivative samples. To separate the derivatives, a DB-5MS fused-silica capillary column (30 m × 0.25 mm × 0.25 μm, Agilent J&W Scientific, Folsom, CA, USA) was utilized. A constant flow rate of 1 mL/min of helium (>99.999%) was employed as the carrier gas through the column. The injector temperature was maintained at 260 °C, and the injection volume was 1 μL in splitless mode. Initially, the oven temperature was set at 60 °C for 0.5 min. Subsequently, it was raised to 125 °C at a rate of 8 °C/min, then increased to 210 °C at a rate of 4 °C/min, followed by a further increase to 270 °C at a rate of 5 °C/min. Finally, the temperature was held at 305 °C for 3 min. The MS quadrupole and ion source (electron impact) were set to 150 °C and 230 °C, respectively. A collision energy of 70 eV was applied. Mass data was acquired in a full-scan mode within the *m*/*z* range of 50–500, with a solvent delay time of 5 min. To assess repeatability, quality control samples (QCs) were injected at regular intervals, specifically every 10 samples, throughout the analytical run. This provided a set of data for evaluating the consistency of the analysis.

### 2.11 Statistical analysis

All experimental data were presented as mean ± standard deviation (SD) from a minimum of three replicates. Statistical significance between the datasets was evaluated using a *t*-test. GraphPad Prism 9.0 software was utilized for generating all figures.

## 3 Results

### 3.1 Heat stress inhibited growth of mycelia in *L. edodes*

The effect of heat stress on *L. edodes* mycelia morphology was investigated by growing mycelia at 25 °C for 24 h. Heat stress substantially inhibited mycelia growth in both Le023 and Le023M (Fig. 1B). Mycelia were dense and had more branches prior to heat stress (Fig. 1C). Mycelia became sparse after heat stress and formed a visible heat stress circle. Microstructure study revealed that after exposure to heat stress, mycelia branches were diminished in both strains Le023 and Le023M (Fig. 1C). Le023M mycelia grew faster than Le023Mycelia when cultivated at 25 °C, at 1.91 ± 0.28 mm/day for Le023 and 4.32 ± 0.19 mm/day for Le023M (Fig. 1D). In two strains, heat stress inhibited mycelia growth. However, once heat stress was removed, Le023M mycelia resumed growth at a rate of 1.62 ± 0.22 mm/day for Le023M and 0.88 ± 0.11 mm/day for Le023. When mycelia were exposed to 39 °C for varying periods of time, Le023Mycelia died after 12 h, whereas Le023M did not cease developing until 24 h (Fig. S2). This data suggested that both strains were impacted by heat stress, but mycelia of Le023M recovered quicker when heat stress was removed.

### 3.2 Effect on lipid peroxidation and non-enzymatic antioxidants

**Fig. 2:**
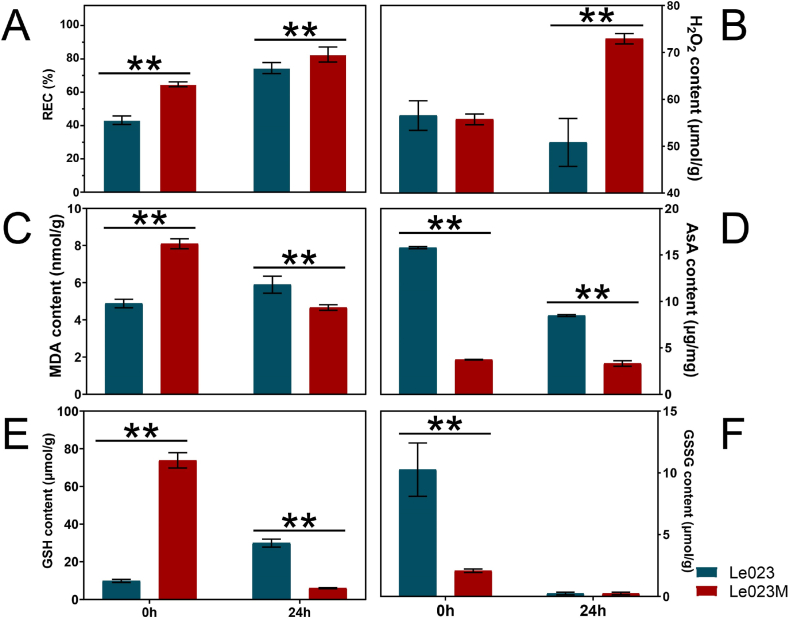
Physiological parameters of mycelia during heat stress. (A) Relative electrolytic conductivity (REC) of mycelia under heat stress. The content of superoxide (B), MDA (C), AsA (D), GHS (E), and GSSH (F) in mycelia under heat stress. Three biological replicates were conducted for each measurement. Asterisks indicate significant differences between the two strains (**p < 0.01).

The cell membrane may be damaged by high temperatures. Heat shock improved mycelia REC in both the Le023 and Le023M strains (Fig. 2A). H_2_O_2_ was accumulated in Le023M during heat stress, but there was a slight decrease in Le023 (Fig. 2B). MDA content in Le023M was greater than in Le023 without heat stress. MDA level in Le023 increased in response to heat stress, but decreased in Le023M (Fig. 2C). Heat stress caused a significant drop in AsA content in Le023. In contrast of Le023, Le023M showed a lower level of AsA with or without heat stress (Fig. 2D). GSH level in Le023 increased swiftly in response to heat stress. Without heat stress, Le023M has a comparatively high amount of GSH compared to Le023. GSH content decreased dramatically after heat stress (Fig. 2E). Le023 showed an extremely high GSSH concentration prior to heat stress. Le023 and Le023M demonstrated a drop in GSSG content after heat stress and maintained a very low level (Fig. 2F).

### 3.3 Differentially expressed genes (DEGs) under heat stress

The differentially expressed gene analysis was conducted to investigate the response to heat stress in the mycelia of Le023 and Le023M strains using transcriptome sequencing on the Illumina Hiseq-2000 system. The transcriptome profiles of Le023 and Le023M strains' mycelia were analyzed after subjecting them to 37 °C for 0 and 24 h. Following the removal of adapter sequences and low-quality reads, an average of 48,070,608 clean reads and 7,031,504,121 clean bases were obtained (Table 1). The average percentage of valid bases and Q30 scores (Phred score ≥ 30) were 95.96% and 94.46%, respectively. By aligning the clean data without a reference genome, a total of 16,139 unigenes with a combined length of 28,076,978 bp were identified.

**Table 1:** RNA-seq data summary and de novo transcriptome assembly statistics for Le023 and Le023M.

Sample	clean_reads	clean_bases	valid_bases	Q30	GC
Le023 0 h	48057706	7034013881	94.93%	94.36%	48.95%
Le023 24 h	47988430	7023711031	94.94%	94.37%	48.84%
Le023M 0 h	48320358	7074662299	95.10%	94.56%	49.06%
Le023M 24 h	47915940	6993629271	94.88%	94.52%	48.81%

**Fig. 3:**
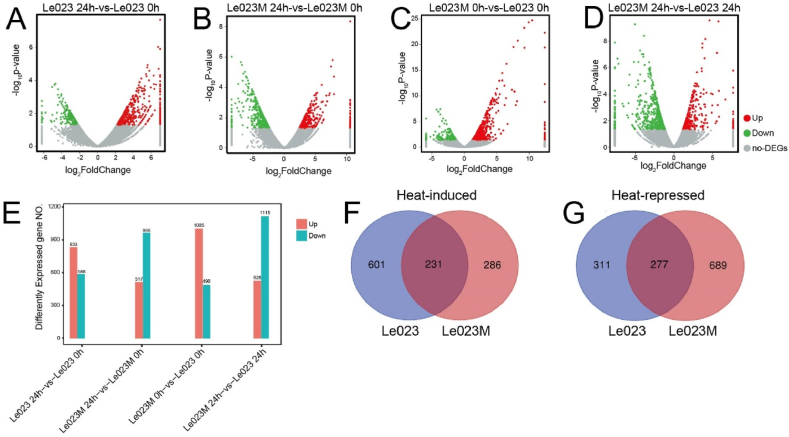
Analysis of Differentially expressed genes (DEGs) between two strains in response to heat stress. Volcano plots of DEGs in different comparisons: (A) Le023 24 h versus Le023 0 h; (B) Le023M 24 h versus Le023M 0 h; (C) Le023M 0 h versus Le023 0 h; (D) Le023M 24 h versus Le023M 24 h. In the volcano plots, red, green, and gray dots represent upregulated genes, downregulated genes, and genes without significant difference, respectively. (E) Column diagram of DEGs in different comparisons. Pink and azure column represent upregulated and downregulated genes, respectively. Y-axis indicates the number of genes. Venn graph for Le023 and Le023M based on heat-induced genes (F) and heat-repressed genes (G). (For interpretation of the references to color in this figure legend, the reader is referred to the Web version of this article.)

Comparing the gene expression levels to the control (0 h of heat stress), a total of 1421 unigenes in the Le023 strain showed significant differential expression (more than two-fold) under heat stress (Fig. 3A and E). Among these DEGs, 833 were upregulated, while 588 were downregulated in response to heat stress (Fig. 3E). Similarly, in the Le023M strain, a total of 1483 DEGs were identified under heat stress (Fig. 3B and E), with 517 unigenes upregulated and 966 unigenes downregulated (Fig. 3E). Importantly, significant differential expression was observed between the Le023 and Le023M strains, with 1495 and 1643 unigenes showing differential expression in the absence or presence of heat stress, respectively (Fig. 3C–E). Following the heat stress, a total of 231 heat-induced DEGs and 277 heat-repressed DEGs were observed in both Le023 and Le023M strains (Fig. 3F and G). These results suggest that the Le023 and Le023M strains display distinct responses to heat shock.

### 3.4 Functional annotation of DEGs under heat stress

**Fig. 4:**
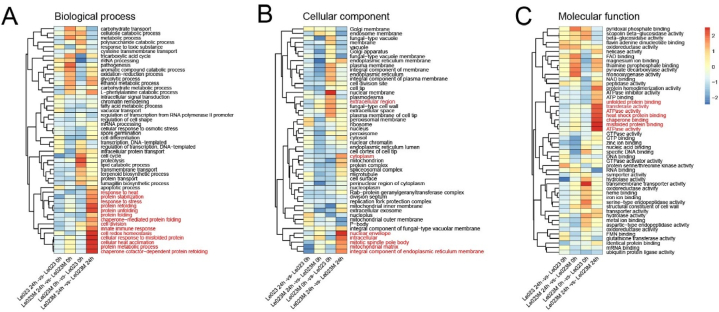
Heatmap of genes involved in different Gene Ontology (GO) classifications. Heatmap showing genes involved in Biological process (A), Cellular component (B) and Molecular function (C). In the heatmaps, red indicates a higher number of genes associated with a specific GO classification, while blue indicates a lower number of genes associated with that GO classification. (For interpretation of the references to color in this figure legend, the reader is referred to the Web version of this article.)

The function of DEGs was investigated by performing Gene Ontology (GO) annotation. GO terms related to biological process (903 terms), cellular component (236 terms), and molecular function (496 terms) were used to compare the responses of Le023 and Le023M strains under heat stress. The top 50 GO terms were analyzed in detail, based on the number of DEGs associated with each term (Fig. 4). In the category of biological process, the majority of DEGs were annotated with terms such as “protein refolding” (P = 7.25^−26^), “protein unfolding” (P = 7.05^−25^), “protein folding” (P = 3.95^−11^), and “response to heat” (P = 7.75^−5^), comprising 51, 48, 45, and 13 DEGs, respectively (Fig. 4A). Regarding cellular components, DEGs were involved in “cytoplasm” (P = 4.52^−4^), “extracellular region” (P = 1.98^−5^), “mitochondrial matrix” (P = 2.13^−4^), and “nuclear envelope” (P = 1.50^−5^), with 221, 73, 29, and 27 DEGs, respectively (Fig. 4B). In the molecular function category, GO terms such as “ATP binding” (P = 9.77^−4^), “ATPase activity” (P = 7.23^−17^), “unfolded protein binding” (P = 3.30^−18^), and “heat shock protein binding” (P = 1.90^−19^) were enriched, with 171, 70, 60, and 36 DEGs, respectively (Fig. 4C). These results indicate that under heat stress, the expression of genes encoding heat shock proteins, which play a role in protein folding and protection against high temperatures, is increased.

### 3.5 Validation of the RNA-seq results by qRT-PCR

**Fig. 5:**
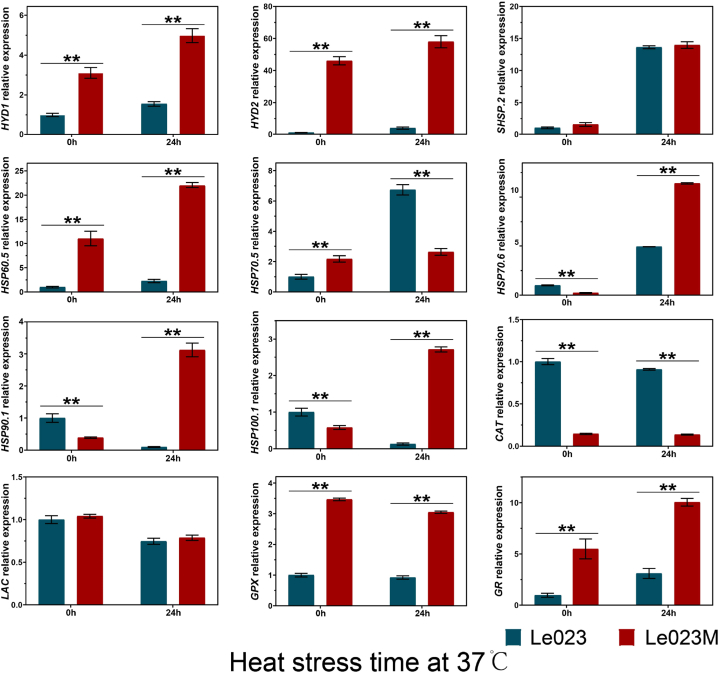
Expression analysis of genes in response to heat stress using qRT-PCR. Relative expression levels of *HYD1*, *HYD2*, *SHSP.2*, *HSP60.5*, *HSP70.5*, *HSP70.6*, *HSP90.1*, *HSP100.1*, *CTA*, *LAC*, *GPX,* and *GR* in response to heat stress were measured. Significant differences between the two strains are indicated by asterisks (**p < 0.01).

By examining the expression patterns of 12 genes associated with heat responses, the validity of the RNA-seq results was confirmed using qRT-PCR (Fig. 5). In response to heat stress, several genes, including *HYD1*, *HYD2*, *HSP60.5*, *HSP70.6*, *HSP90.1*, and *HSP100.1*, were strongly expressed, according to the results of RNA-seq and qRT-PCR study. Under heat stress, the genes that encode GR and GPX were elevated.

### 3.6 Metabolomic response of *L. edodes* under heat stress

**Fig. 6:**
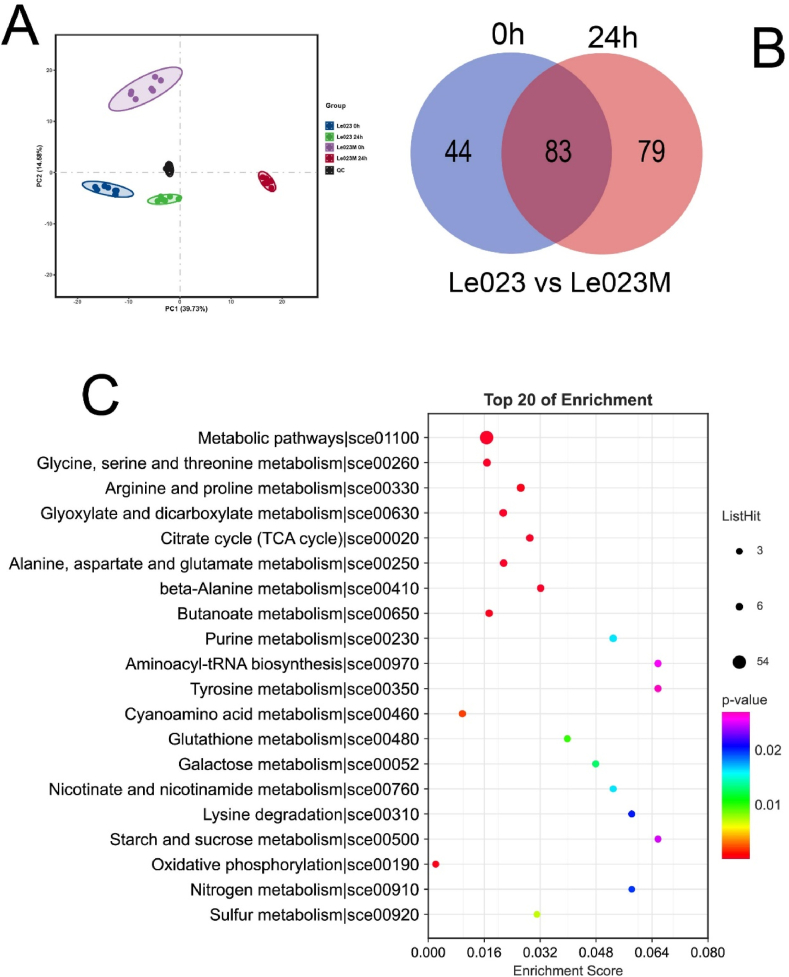
Metabolic analysis of mycelia in response to heat stress. (A) Principal component analysis (PCA) of metabolite profiles in response to heat stress. (B) Venn diagram showing differential accumulated metabolites between Le023 and Le023M under heat stress. (C) Top 20 enriched pathways for overlapping heat-responsive genes between Le023 and Le023M. The size of the circle represents the number of metabolites and the color bar indicates the significance of enrichment. (For interpretation of the references to color in this figure legend, the reader is referred to the Web version of this article.)

By employing unbiased metabolite profiling using GC-MS and generating a data matrix, we were able to investigate the impact of heat stress on the overall metabolism of Le023 and Le023M strains. Principal component analysis (PCA) was conducted to reduce the dimensionality of the data matrix (Fig. 6A). The clustering of the dark spots (QC) indicated the reliability of the data matrix for further analysis. Through this approach, a total of 297 metabolites were systematically identified under different treatments. Remarkably, 162 metabolites exhibited significant differences between Le023 and Le023M when exposed to heat stress (Fig. 6B and Table S2). Among these, 86 metabolites showed a significant decrease in Le023M compared to Le023, while 76 metabolites exhibited a significant increase (Table S2). Pathway enrichment analysis was employed to investigate the metabolic pathways associated with these 162 differential metabolites (Fig. 6C). The results revealed that these metabolites are primarily involved in metabolic pathways, citrate cycle, sugar metabolism, and amino acid metabolism.

### 3.7 Targeted metabolomic analysis of key metabolites

**Fig. 7:**
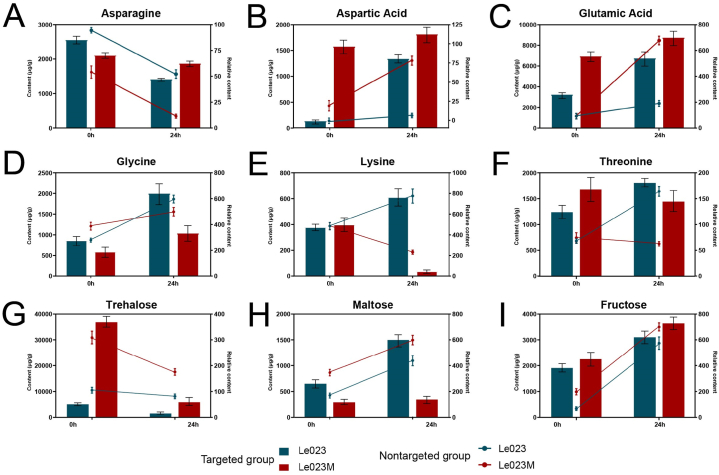
Targeted metabolic analysis of nine metabolites in response to heat stress. The analyses based on targeted GC-MS are shown as columns (left Y-axis). The relative expression levels of metabolites determined by non-targeted GC-MS analysis are represented by lines (right Y-axis). The values in the columns are presented as the means ± SD of seven independent experiments.

Six types of amino acid or amino acid derivatives and three types of soluble sugars were selected for targeted verification (Fig. 7). The overall trend of the measured targeted content significantly corresponded with the relative content of metabolites. Upon exposure to heat stress, the levels of asparagine and trehalose in both Le023 and Le023M mycelia decreased (Fig. 7A and G). Interestingly, prior to heat stress, trehalose content remained relatively elevated in Le023M. Following heat stress, there was an increase in the levels of aspartic acid, glutamic acid, glycine, maltose, and fructose in both Le023 and Le023M strains (Fig. 7B–D, H and I). Notably, Le023 and Le023M exhibited contrasting accumulation patterns in lysine and threonine, with Le023Maintaining relatively high levels of lysine and threonine after heat stress (Fig. 7E and F). These findings indicate that metabolomic analysis can properly reflect the metabolic deviations in mycelia’s response to heat stress.

### 3.8 Transcriptomic and metabolic regulatory pathways of *L. edodes* under heat stress

**Fig. 8:**
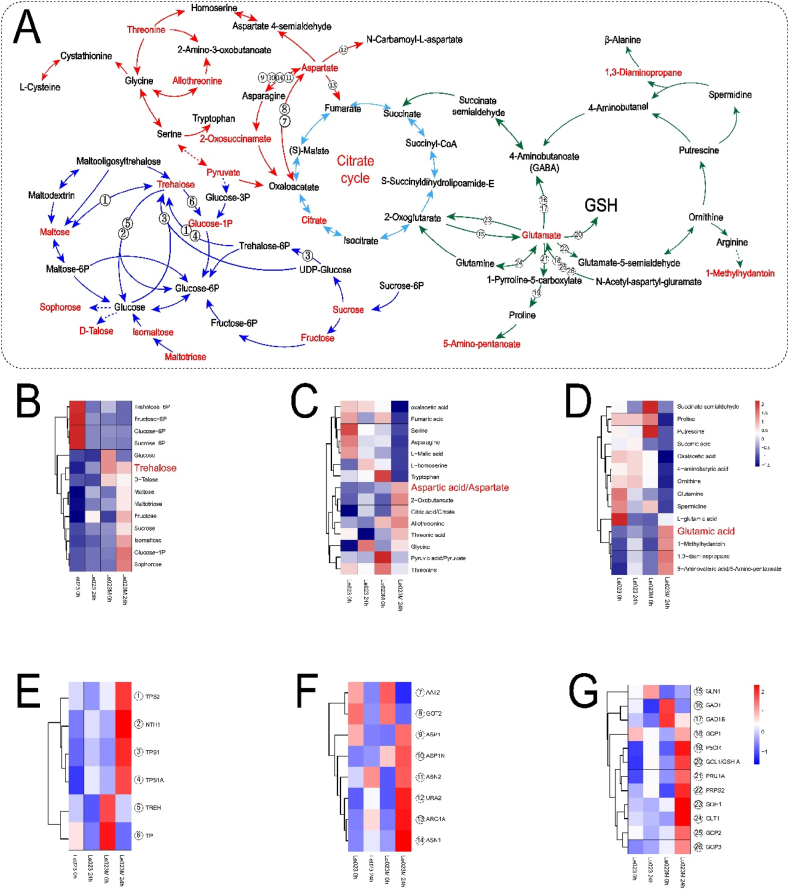
A flow diagram showing differential metabolite accumulation in response to heat stress. (A) Metabolism of trehalose, aspartate, and glutamate in the mycelia of Le023 and Le023M in response to heat stress. Red front indicates metabolites are significantly accumulated in Le023M in response to heat stress. Heatmap of metabolites related to sugar (B), aspartate (C) and glutamate (D) metabolism. Heatmap of expressed genes involved in trehalose (E), aspartate (F) and glutamate (G) metabolism. (For interpretation of the references to color in this figure legend, the reader is referred to the Web version of this article.)

Enrichment analysis of differential metabolites revealed the predominant involvement of the citrate cycle, starch and sucrose metabolism, and amino acid metabolism pathways in the response to heat stress in both Le023 and Le023M strains (Fig. 8A). Specifically, within the starch and sucrose metabolism pathways, we identified 14 metabolites that showed significant changes in response to heat stress in Le023 and Le023M (Fig. 8B). Upon heat stress, nine metabolites, including trehalose, d-talose, and maltose, accumulated in Le023M. In contrast, trehalose-6-phosphate, fructose-6-phosphate, glucose-6-phosphate, and glucose were highly accumulated in Le023 prior to heat stress. Gene expression analysis revealed the upregulation of genes involved in trehalose metabolism in Le023M under heat stress (Fig. 8E). To investigate the impact of trehalose on mycelial growth, exogenous trehalose was introduced, resulting in enhanced tolerance of both Le023 and Le023M mycelia to heat stress (Fig. S3). In the glycine, serine, and threonine metabolism pathways, Le023M showed relatively high accumulations of aspartic acid, 2-oxobutanoate, l-allothreonine, threonic acid, and glycine after heat stress (Fig. 8C). Transcription of genes involved in aspartate metabolism was significantly upregulated in Le023M under heat stress (Fig. 8F). On the other hand, Le023M exhibited higher accumulations of glutamic acid, 1-methylhydantoin, 1,3-diaminopropane, and 5-aminovaleric acid under heat stress, while Le023 showed increased levels of succinate semialdehyde, proline, and putrescine without heat stress (Fig. 8D). Gene expression profiles demonstrated the upregulation of genes involved in glutamic acid metabolism in Le023M in response to heat stress (Fig. 8G). The upregulation of these genes suggests that Le023M exhibits an adaptive response to heat stress. The results suggest that Le023M has a higher capacity to survive heat stress than Le023.

## 4 Discussion

With a growing global population, the amounts of CO_2_ and other greenhouse gases are dramatically increasing, resulting in global warming. The shiitake mushroom is a hypothermic and fruiting mushroom, which requires a temperature of 24–27 °C for mycelium growth and 10–12 °C for primordium differentiation. Heat stress can inhibit the growth of mycelia, thus decreasing the yield and inducing a negative effect on mushroom quality. High temperature could damage the integrity of the cell wall and cell membrane, thereby causing functional changes by altering the structure of plasma membrane, such as the loss of permselectivity [30].

### 4.1 Heat stress effects on mycelia growth and cellular responses

Heat stress is known to have a significant impact on the growth of *L. edodes* mycelia. The results showed that heat stress substantially inhibited mycelia growth in both Le023 and Le023M strains (Fig. 1B). Prior to heat stress, mycelia were dense with more branches, but they became sparse and formed a visible heat stress circle after exposure to heat stress (Fig. 1B and C). Moreover, heat stress inhibited mycelia growth in both strains, with Le023M mycelia recovering faster than Le023Mycelia once heat stress was removed (Fig. 1D). These findings highlight the sensitivity of *L. edodes* mycelia to heat stress and the potential differences in the growth response between different strains. When mycelia were exposed to heat stress, membrane structure was damaged, and permeability was increased, resulting in an increase in REC by exosmotic electrolytes from cells [31]. Increased REC reduced the cell''s ability to maintain homeostasis and caused cell death. High temperature can damage the cell membrane, and REC can be selected as an indicator to evaluate the degree of damage [32]. After heat stress, the REC of these two strains improved a lot, reaching 75.1% and 83.3%, respectively, indicating that the basic selective permeability of the cell membrane was lost (Fig. 2A). A higher relative conductivity was found for strain Le023M than for strain Le023, indicating a high heat sensitivity. Le023M responded very quickly to high temperature stress due to its sensitivity.

Reactive oxygen species (ROS) not only play critical roles in maintaining normal plant growth but also contribute to disease resistance [33]. Under normal conditions, cells maintain a balance between ROS formation and elimination. However, heat stress disrupts this delicate balance, resulting in an increase in ROS concentrations. The extent of membrane peroxidation can be indicated by the levels of malondialdehyde (MDA) [34]. Our findings align with previous research [35], as we observed an increase in MDA levels with prolonged heat stress, indicating more severe membrane oxidation. Remarkably, unlike other studies, Le023M exhibited an increase in MDA concentration with prolonged heat stress (Fig. 2C), suggesting a unique response to heat due to its sensitivity [36].

Under heat stress, the enzymatic system within cells can undergo changes, leading to differences in the response to heat stress between the two strains. Le023M may exhibit higher enzyme activity, which helps it maintain normal metabolic activity and growth under high temperature. These enzymes may include antioxidant enzymes such as glutathione (GSH and GSSH) and AsA, which can eliminate harmful oxygen radicals produced within cells and alleviate oxidative stress-induced damage [37]. Prior to heat stress, the accumulation of AsA in Le023M was reduced as compared to Le023. Under heat stress, it was shown that both strains progressively degraded AsA to scavenge ROS (Fig. 2D). In the antioxidant defense system, GSH plays a key role in the removal of ROS, methylglyoxal (MG), and endogenous toxic compounds by acting as a substrate or cofactor of GPX, glutathione S-transferase (GST), and glyoxalase I (GLy I). Prior to heat stress, Le023M accumulated more GSH than Le023 (Fig. 2E). After heat stress, mycelia began to consume GSH and GSSH to remove ROS, and the high content of GSH in Le023M made it more efficient at removing ROS at high temperature. The expression levels of GSH and GSSG were upregulated in response to heat stress, thus promoting the synthesis of GSH and GSSG, which were used for eliminating the damage of cell membrane by superoxide. In addition, other enzymes such as heat shock proteins may also be involved in the response to heat stress, assisting in maintaining cellular stability and function.

### 4.2 Heat stress induces upregulation of heat shock proteins (HSPs) in Le023M mycelia

Under abiotic stress, HSPs function as chaperones involved in normal cellular processes, including protein folding, assembly, translocation, and degradation. According to GO analysis, the DEGs were enriched in “response to heat”, “response to stress”, “protein folding”, and “chaperonin” in response to heat stress (Fig. 3). We found that many *LeHsp* genes, including *HSP60.5*, *HSP70.6*, *HSP90.1*, and *HSP100.1*, were strongly up-regulated after heat stress in Le023M. This finding supports the idea that the over-expression of *Hsp70* genes could enhance the capacity of plants to cope with high temperature [[38], [39], [40]]. When the mycelia were exposed to heat stress, the synthesis of HSP70 forms a central hub of molecular chaperone networks with other HSPs to prevent the aggregation and misfolding of proteins, thereby facilitating the folding and modification of newly synthesized proteins or preventing programmed cell death under stress by inhibiting the activity of telomerase involved in signal transduction pathway [[41], [42], [43]]. Heat shock transcription factors (HSFs) were first activated by heat signal and then regulated *HSPs* expression when cells were subjected to heat stress [44]. Transcription of heat shock protein genes accumulated in response to high temperature, drought, oxidative, chemical toxicity, and other stresses [45,46], and heat tolerance was strongly correlated with the expression of heat shock protein genes [47]. The expression of genes like *HSP60.5*, *HSP70.6*, *HSP90.1*, and *HSP100.1* were significantly increased in Le023M following 24 h of heat stress compared to Le023, suggesting that these genes are essential for mycelia’s ability to survive heat, in a finding consistent with the fact that heat shock protein genes are positively correlated with thermotolerance and enhance plant and fungi tolerance to salt, water, and high temperatures [45,48].

### 4.3 Role of trehalose, glutamate, and aspartate in heat stress tolerance of Le023M mycelia

Trehalose plays a key role in the tolerance to abiotic stress such as high salt, low pH, and high temperature [[49], [50], [51], [52]]. The accumulation of trehalose in the Botrytis cinerea *Δtps1* mutant demonstrated improved tolerance at high temperature in the presence of abiotic stress [53]. In this study, exogenous trehalose treatment in Le023 suggested that trehalose could improve mycelia’s tolerance to high temperature (Fig. S3). Trehalose enhances the tolerance to heat in cells via different metabolic pathways. Exogenous trehalose in *P. ostreatus* demonstrated that glycolysis was inhibited, and pentose phosphate pathway was stimulated to enhance heat tolerance [54]. Trehalose plays an essential role in the ROS scavenging mechanism when exposed to high temperature in cells [55,56]. The ability of *S. cerevisiae* to cope with H_2_O_2_ stress may be increased through treatment with 10% trehalose [57]. When studying antioxidant system of wheat callus under water stress, Ma, Wang [58] found exogenous trehalose improved the resistance to heat stress, but it could not scavenge ROS, possibly because the protective effect of exogenous trehalose was primarily reflected in improving the activity of antioxidant enzymes and other aspects [58]. In strain Le023M, significant accumulation of trehalose in Le023, which plays a crucial role in heat stress tolerance.

Seeding pretreatment with glutamate (Glu) may improve heat stress tolerance in maize [59]. The level of Glu in the mycelia of Le023M was found to be greater than that of Le023. Under heat stress, the transcripts of genes implicated in the glutamate metabolism pathway were dramatically increased in strain Le023M, showing that Glu played a vital role in heat stress tolerance. The elevated levels of Glu and Asp in buckwheat considerably enhanced the activity of superoxide dismutase and ascorbate peroxidase in eliminating superoxide generated under heat stress [60]. Similarly, exogenous addition of Asp and Glu may improve *A. pasteurianus* acid stress tolerance by increasing pentose phosphate and NADPH production [61]. The accumulation of Asp and Glu in the mycelia of *L. edodes* may promote the formation of NADPH, a key cofactor in the GSH metabolism pathway that can stimulate GSH synthesis and scavenge ROS caused by heat stress.

Transcriptomic and metabolic analyses under heat stress reveal that the differentially expressed genes were involved in protein folding or refolding. Proteins like HSP70s, which are activated by high temperatures, may repair heat-damaged proteins. Furthermore, the genes associated in the trehalose, glutamate, and aspartate metabolic pathways were substantially expressed in response to heat stress in Le023M. Metabolic study revealed a considerable increase of trehalose, Glu, and Asp in Le023M mycelia. These compounds may improve heat stress tolerance in Le023M mycelia through the GSH metabolism pathway. Le023M’s mycelia may improve its heat tolerance in a variety of ways.

## 5 Conclusion

Heat stress significantly affects the growth and cellular responses of *L. edodes* mycelia. The Le023M strain demonstrates higher sensitivity to heat stress compared to the Le023 strain. Heat stress induces membrane damage, ROS accumulation, and oxidative stress. However, Le023M exhibits a unique response with increased MDA concentration, suggesting its distinct sensitivity to heat stress. The upregulation of HSPs and the involvement of trehalose, glutamate, and aspartate contribute to heat stress tolerance in Le023M mycelia. These findings provide insights into the cellular mechanisms underlying heat stress response in *L. edodes* and may contribute to the development of strategies for enhancing heat tolerance and improving mushroom cultivation practices.

## Funding

This work was supported by Gansu Provincial Youth Science and Technology Fund Program (No. 20JR5RA064), Scientific and Technological Innovation Talents of Sichuan Province (No. 2022JDRC0034), Technology Innovation Guidance Program (No. 22CX8NH219), Central Public-interest Scientific Institution Basal Research Fund (No. S2022007), Local Financial Funds of National Agricultural Science and Technology Center, Chengdu (No. NASC2021KR06), Liangshan Science and Technology Program and Technology innovation guidance program (22CX8NH219).

## Author contribution statement

Qin Zhang and Xu Zhao: Conceived and designed the experiments Analyzed and interpreted the data and Wrote the paper.

Rencai Feng and Renyun Miao:Contributed reagents, materials, analysis tools or data.

Junbin Lin, Luping Cao, Yanqing Ni, and Wensheng Li: Performed the experiments and Analyzed and interpreted the data.

## Data availability statement

The authors do not have permission to share data.

## Additional information

No additional information is available for this paper.

## Declaration of competing interest

The authors declare that they have no known competing financial interests or personal relationships that could have appeared to influence the work reported in this paper
