# Supplementary material for: Combined transcriptomics and metabolomics analysis reveals the molecular mechanism of heat tolerance of Le023M, a mutant in Lentinulaedodes
Source: Heliyon. 2023 Jul 17;9(7):e18360. doi: 10.1016/j.heliyon.2023.e18360 (PMC10372740; doi:10.1016/j.heliyon.2023.e18360)
Supplement: Multimedia component 1 [file mmc1.docx]

**Supplementary material**


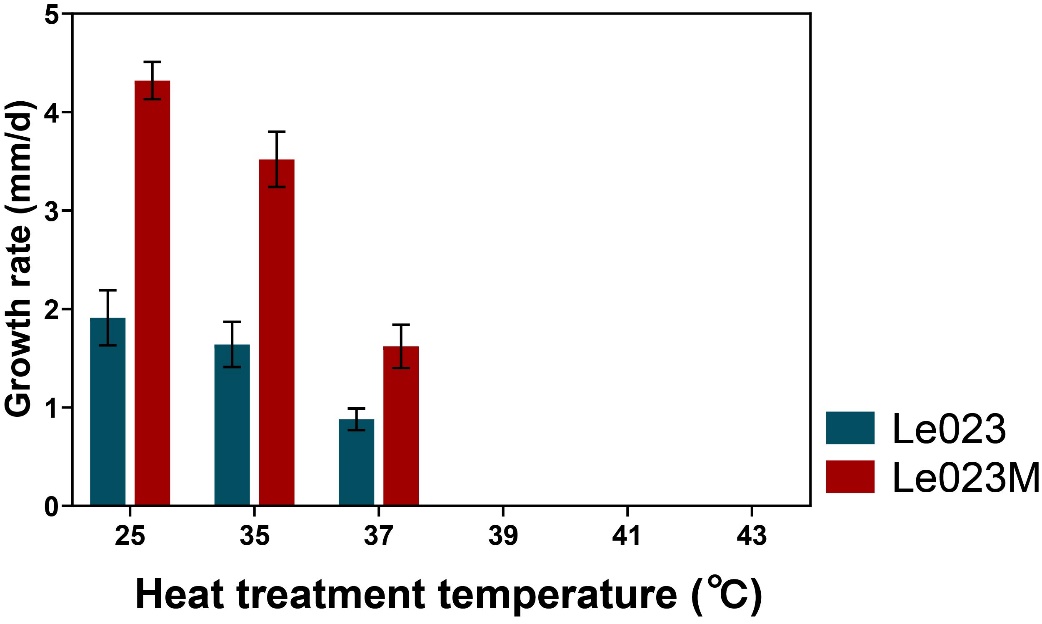


**Figure S1.**Mycelial growth rate at various temperatures for 24 h.

This figure displays the growth rate of mycelia under different temperature conditions for a duration of 24 h.


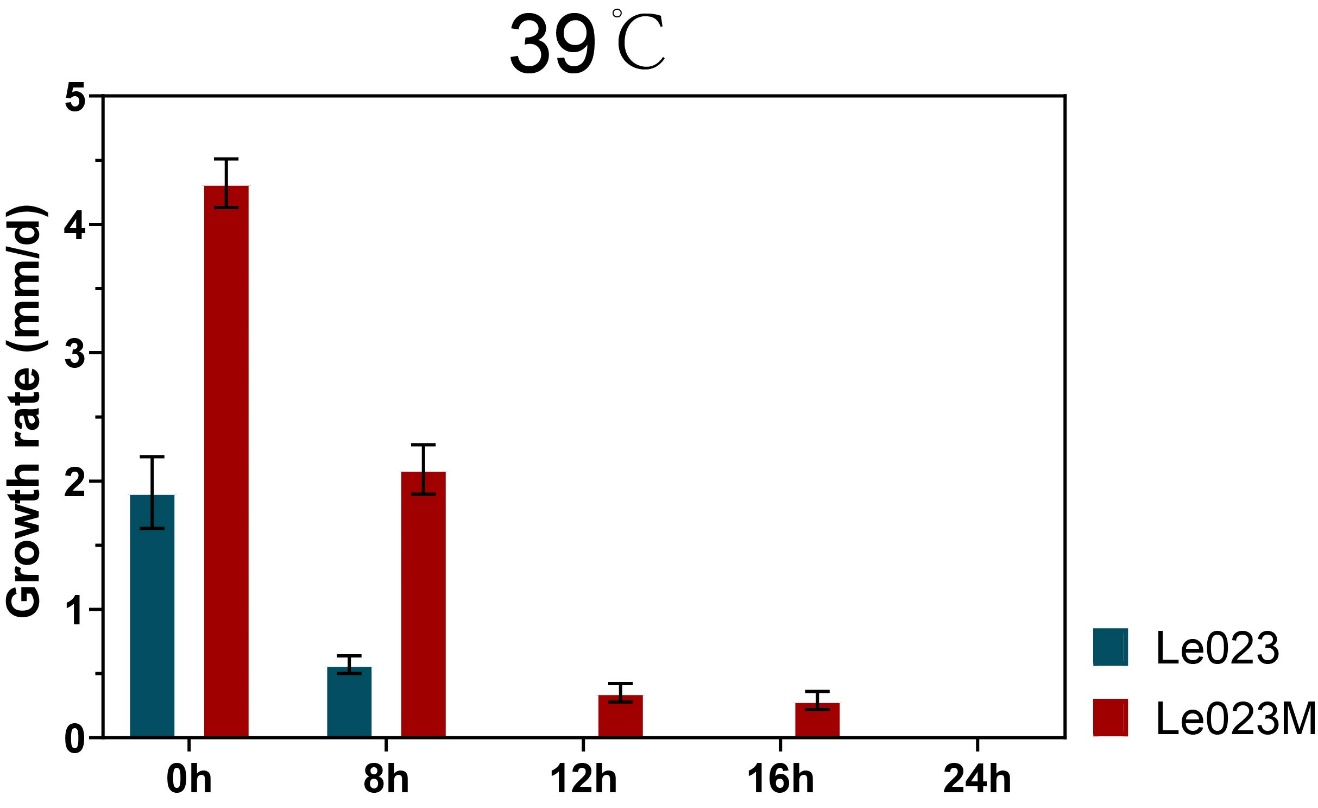


**Figure S2.**Impact of heat stress (39 °C) on mycelial growth rate.

This figure illustrates the influence of heat stress, specifically at a temperature of 39 °C, on the rate of growth.


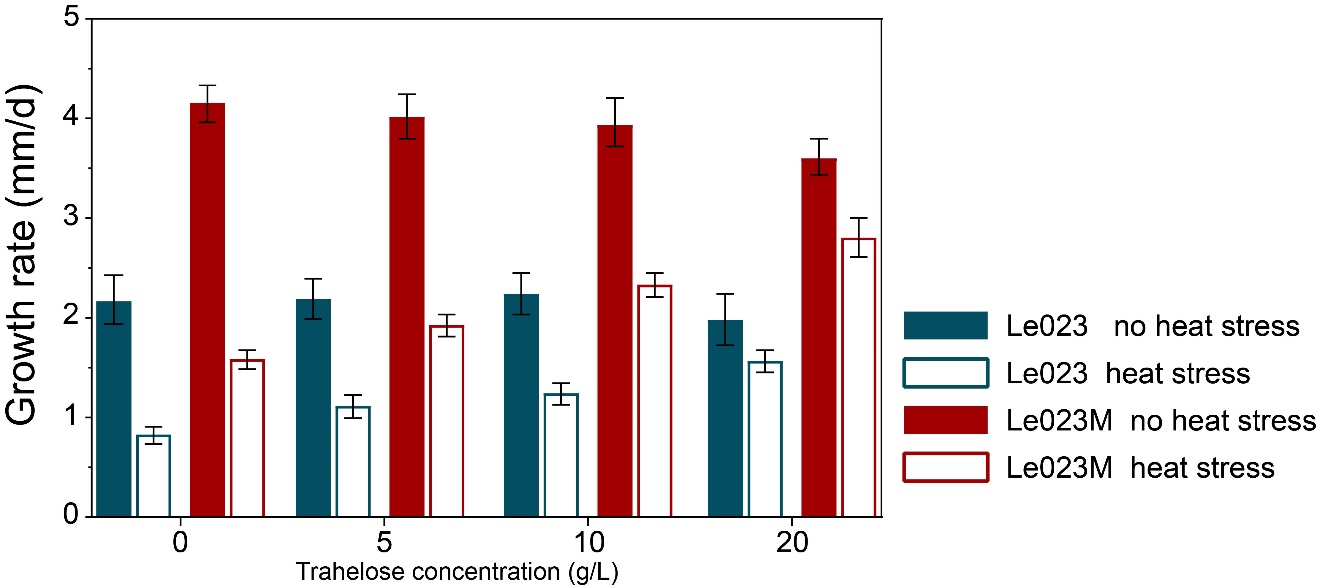


**Figure S3.**Impact of exogenous trehalose on mycelial growth rate.

This figure demonstrates the effect of adding exogenous trehalose ongrowth rate of mycelia.

**Table S1.**Primer pairs used for gene expression (RT-qPCR).

| Genes | Forward sequence (5’-3’) | Reverse sequence (5’-3’) |
| --- | --- | --- |
| *HYD1* | AATGGAGCGGTGGAGGA | AAGTGAGCCCGACAGGA |
| *HYD2* | TCCTCGGTATCGTCATCC | GTTGTCAGTGCAGCAGA |
| *sHSP.2* | ACCTTTGACTCGCTCACCTG | CATGGTCTCCAACACGGACA |
| *HSP60.5* | GAGCCAGCATTCTGAAGGGT | TTGCTACAGTGACACCGTCC |
| *HSP70.5* | ATCAAGCACTGGCCTTTCGA | TCAGGGGTAAAATCGCGCTT |
| *HSP70.6* | TCCTCGCTCGGAACCTA | GCACCCTGAACCCTTTT |
| *HSP90.1* | TACTCCGCATACTTGGTTG | GAGGGTTGACAGTGTCCTG |
| *HSP100.1* | CATCGTCAAGCGGGCAC | TGGGCAATGTAAGAATCGTG |
| *CAT* | GGTGGTGCTCGAGACTTTGA | TCACGCTGTTCGTCATTCCA |
| *LAC* | TTCACTTGCACGGACACCAT | TGTCCTTTGGCTGGGATGAC |
| *GR* | GAGAACATCCCAACCGTCGT | GTTCTTCCTCGACCATCGCA |
| *GPX* | GCTTCAACCACCACTCCTCA | GGAACACCAACCCCGGATAC |
| *TUB* | GACATTTGCTTCCGAACCCT | CGGACATAACAAGGGACACA |

**Table S2.** Identification of differential metabolites

| Metabolites | Le023 | | Le023M | |
| --- | --- | --- | --- | --- |
|  | 0 h | 24 h | 0 h | 24 h |
| Sophorose | 52.14±9.5 | 198.2±30.82 | 206.9±53.91 | 1318.19±107.33 |
| Oxalic acid | 19.95±14.75 | 163.49±43.83 | 218.34±66.11 | 987.4±67.86 |
| Maltose | 171.41±30.43 | 439.07±59.97 | 345.73±81.78 | 599.09±31.57 |
| D-Talose | 19.86±48.65 | 11.62±30.73 | 177.68±94.59 | 147.76±106.4 |
| Fructose | 90.96±16.16 | 580.78±45.38 | 215.7±34.89 | 703.74±38.7 |
| 1,3-diaminopropane | 39.9±3.58 | 102.98±6.82 | 59.84±10.79 | 200.04±7.58 |
| Glucose-1-P | 7.84±3.33 | 14.9±2.57 | 16.7±2.96 | 79.04±6.45 |
| 5-Methoxytryptamine | 32.6±2.05 | 78.8±4.25 | 56.3±9.16 | 129.22±3.77 |
| 8-Aminocaprylic acid | 2.05±0.26 | 12.07±1.16 | 10.77±3.11 | 46.7±3.32 |
| Galactose | 0.39±0.14 | 0.65±0.13 | 7.51±3.69 | 30.09±4.52 |
| Tyrosine | 20.71±2 | 48.53±5.25 | 28.67±4.55 | 76.79±3.31 |
| Isomaltose | 7.54±1.01 | 10.31±1.06 | 14.14±1.78 | 33.84±1.86 |
| Phenylalanine | 65.65±8 | 48.06±3.71 | 144.99±10.54 | 69.85±4.81 |
| Gallic acid | 6.1±0.76 | 14.04±1.91 | 8.92±1.87 | 28.08±0.84 |
| Leucrose | 0.11±0.26 | 0.08±0.21 | 2.54±0.35 | 9.83±0.91 |
| Guanosine | 0.87±0.29 | 1.99±0.37 | 6.64±0.73 | 7.3±0.17 |
| Palatinitol | 1.97±0.89 | 0.78±0.29 | 40.59±7.72 | 4.43±1.43 |
| 2-hydroxypyridine | 7.73±0.82 | 9.88±0.93 | 5.71±0.89 | 13.18±0.86 |
| 5-Aminovaleric acid | 1.97±0.13 | 4.63±0.26 | 2.96±0.58 | 7.08±0.4 |
| β-Mannosylglycerate | 11.31±2.02 | 4.25±0.62 | 6.73±1.06 | 6.64±0.35 |
| Allylmalonic acid | 0.62±0.07 | 1.38±0.09 | 1.27±0.25 | 3.75±0.26 |
| Acetol | 0.76±0.11 | 2.38±0.23 | 1.3±0.27 | 4.73±0.19 |
| Sorbitol | 3.6±0.34 | 2.02±0.35 | 0.65±0.06 | 3.8±0.27 |
| Gly-pro | 2.14±0.15 | 3.82±0.22 | 7.89±0.65 | 5.03±0.19 |
| Octanal | 0.21±0.03 | 0.61±0.06 | 0.38±0.09 | 1.04±0.04 |
| 21-hydroxypregnenolone | 0.06±0.01 | 0.25±0.08 | 0.13±0.03 | 0.48±0.08 |
| Citraconic acid | 0.5±0.14 | 0.47±0.06 | 0.39±0.14 | 0.54±0.11 |
| Atrazine-2-hydroxy | 0.01±0.01 | 0.02±0 | 0 | 0 |
| 2-Monopalmitin | 2.58±1.02 | 0.07±0.02 | 0.31±0.15 | 0 |
| 3,5-Dihydroxyphenylglycine | 0.01±0.03 | 0.09±0.07 | 0.09±0.01 | 0 |
| 2-ketoadipate | 0.4±0.06 | 0.48±0.03 | 0 | 0 |
| D-Fructose 1,6-bisphosphate | 9.05±4.77 | 0.57±0.24 | 1.73±0.36 | 0 |
| Adenine | 1.61±0.22 | 0.66±0.03 | 0.91±0.14 | 0 |
| Fructose-6-P | 7.03±2.62 | 0.72±0.38 | 0.37±0.28 | 0 |
| Ethyl cinnamate | 0.51±0.04 | 1±0.1 | 0 | 0 |
| α-ketoglutaric acid | 6.48±1.42 | 1.73±0.19 | 1.71±0.93 | 0.18±0.04 |
| 1,5-Anhydroglucitol | 2.61±0.22 | 1.88±0.44 | 0.92±0.38 | 0.14±0.02 |
| (2R)-2-amino-3-phosphonopropanoic acid | 1.69±0.37 | 1.88±1.65 | 0.39±0.07 | 0 |
| Nicotinic acid | 6.28±0.53 | 4.56±0.44 | 1.82±0.18 | 2.15±0.09 |
| D-Glyceric acid | 8.35±3.9 | 4.28±0.17 | 1.58±0.19 | 1.37±0.07 |
| Creatine | 9.32±1.48 | 7.21±0.95 | 5.93±1.09 | 3.81±0.36 |
| N-Acetyl-D-Galactosamine | 9.99±1.73 | 4.49±1.33 | 19.24±1.79 | 0.62±0.29 |
| Pyrophosphate | 4.45±3.8 | 7.38±4.58 | 14.53±2.9 | 0 |
| Succinic acid | 8.22±1.62 | 9.43±1.32 | 2.21±0.75 | 0.61±0.07 |
| Gluconic acid | 25.53±4.4 | 25.41±6.76 | 8.14±4.3 | 1.88±0.21 |
| Asparagine | 96.76±10.28 | 52.08±6.31 | 53.95±12.97 | 11.33±1.94 |
| Threitol | 49.01±6.57 | 51.24±11.25 | 4.87±0.91 | 0.74±0.23 |
| L-Malic acid | 115.37±20.87 | 64.53±5.98 | 43.18±13.31 | 4.16±1.02 |
| Serine | 307.82±23.27 | 225.72±27.3 | 202.65±56.56 | 148.74±10.24 |
| Ethanolamine | 38.47±12.13 | 141.64±25.82 | 64.46±17.67 | 9.1±3.24 |
| D-glycerol 1-P | 156.72±35.8 | 154.18±21.84 | 86.06±9.42 | 9.86±2.46 |
